# Pediatric Repetitive Mild Traumatic Brain Injury Elicits T Cell-Mediated Neuroinflammation

**DOI:** 10.1177/2689288X251400587

**Published:** 2026-01-28

**Authors:** Kirill Shumilov, Lindsay Arellano, Yongyun Shin, Grace Dominguez, Rajiv Swarup, William Bernhardt, John Greer, James P. McAllister, Maria Garcia-Bonilla, Kimberle Jacobs, David D. Limbrick

**Affiliations:** 1 Department of Neurosurgery, Virginia Commonwealth University, Richmond, Virginia, USA.; 2 Department of Biostatistics, Virginia Commonwealth University, Richmond, Virginia, USA.; 3 Department of Anatomy and Neurobiology, Virginia Commonwealth University, Richmond, Virginia, USA.

**Keywords:** immune cell infiltration, neuroinflammation, pediatric repetitive traumatic brain injury, T cells

## Abstract

Pediatric repetitive mild traumatic brain injury (rmTBI) is a major public health concern with links to chronic cognitive dysfunction. Neuroinflammation represents a significant maladaptive outcome after rmTBI. Persistent innate and adaptive immune cell responses can lead to neurodegeneration and deficits in brain development. Therefore, a deeper understanding of the early dynamics of peripheral immune cell infiltration after pediatric rmTBI is critical for the development of effective treatment. We hypothesize that pediatric rmTBI alters neuroinflammation through T cell infiltration. We used wild-type (C57BL/6) and T cell knockout (TCRβ^−/−^ δ^−/−^) mice to test this hypothesis. We developed a pediatric postnatal day 21 rmTBI model, with three consecutive subconcussive impact acceleration injuries separated in time by 1 week. After inducing rmTBI in juvenile mice, we observed a progressive infiltration of macrophages, CD8^+^, and CD4^+^ T cells into the brain parenchyma, which increased with repeated injury. Furthermore, neuroinflammation in the white matter was detected when we analyzed the lateral corpus callosum (CC). Since increased infiltration of CD4^+^ and CD8^+^ T cells was detected, we utilized TCRβ^−/−^ δ^−/−^ mice to further explore the role of T cells on neuroinflammation after pediatric rmTBI. We observed a reduction in the infiltration of pro-inflammatory macrophages and decreased neuroinflammation in the lateral CC. Overall, our findings highlight the significant role of T cell infiltration in the modulation of neuroinflammation following rmTBI in the developing brain, suggesting that they may serve as potential therapeutic targets for managing neuroinflammation following pediatric brain injuries.

**Figure:**
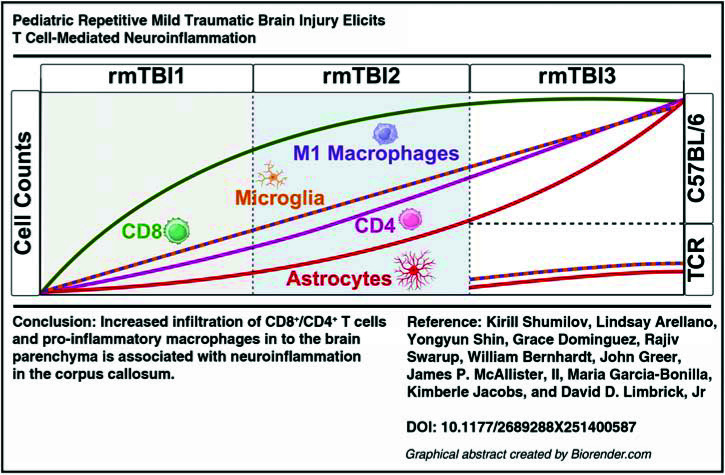


## Introduction

Mild traumatic brain injury (mTBI) accounts for 80% of all traumatic brain injury (TBI) cases and is the main cause of disability and neurological morbidity in children.^1^ TBI-related emergency room visits have increased alarmingly among pediatric patients, with the rate rising from 1374 per 100,000 in 2007–2008 to 2194 per 100,000 in the 0–4 years age group in 2009–2010.^2,3^ Pediatric TBI differs substantially from adult TBI due to age-specific biomechanics and differences in pathophysiology. In part, these differences result from the dynamic developmental landscape affecting cellular programming, brain structure, vascularization, and regulation.^4–7^ Despite the high incidence of pediatric TBI, the exact mechanisms by which rmTBI leads to poor neurological outcomes remain unclear. This lack of understanding hinders the development of effective treatments and optimal clinical management. Therefore, it is crucial to elucidate the underlying mechanisms of rmTBI to prevent long-term neurological and neurobehavioral issues.

Although the developing brain is often considered for its capacity for plasticity, it is also particularly susceptible to long-term consequences from early-life TBI. Accordingly, clinical studies support the idea that younger children exhibit greater cognitive impairments after TBI and are more likely to develop long-term academic difficulties.^5,8,9^ Pediatric rmTBI pathophysiology has only begun to be explored, and the long-term impact of pediatric neuroinflammation and immune cell infiltration is largely unknown. Previous studies using adult rodents have reported that, during the inflammatory phase after mTBI, the immune system maintains an elevated pro-inflammatory response.^10–12^ This may be related to the continuous presence of peripheral immune cells. For example, elevated lymphocytes have been detected 2 weeks post-injury in juvenile rats, while in adults, inflammation subsided after 7 days.^13,14^ Furthermore, T cells can accumulate in the lesion site and persist for months after central nervous system injury.^15,16^ Nevertheless, the impact of T cells in the pathophysiology of pediatric rmTBI has not been fully explored.

Unraveling the cellular underpinnings of neuroinflammatory sequelae following rmTBI could pave the way for a novel targeted treatment, urgently needed for this neurological condition. Recent advancements in immunotherapy for related brain injuries, such as the depletion of CD4^+^ T cells using specific monoclonal antibodies,^17,18^ highlight the potential of T cell modulation for rmTBI. T cell activation may be particularly important in pediatric patients with TBI. The immune system is crucial for brain development, so even mTBI could lead to immune dysregulation of subsequent brain processes and maturation. Therefore, this dysregulation could result in worse outcomes in pediatric patients compared to adults.^14^ Thus, we hypothesize that pediatric rmTBI increases astrocyte and microglia/macrophage activation through peripheral T cell infiltration. In this article, we used genetic deletion of TCRβ^−/−^ δ^−/−^ (TCR KO) in a juvenile mouse model to understand the overall impact of infiltrating lymphocytes on the neuroinflammatory response in the juvenile brain after three rmTBI spaced 1 week apart. Our data suggest that increased infiltration of CD8^+^/CD4^+^ T cells and pro-inflammatory macrophages into the brain parenchyma is associated with neuroinflammation in the corpus callosum (CC). Therefore, a deeper understanding of the mechanism by which pediatric rmTBI neuroinflammation is modulated by T cells will contribute toward the development of a highly translational targeted immunotherapy for this neuroinflammatory injury.

## Materials and Methods

### Animals

Postnatal day 21 C57BL/6 and TCRβ^−/−^ δ^−/−^ (Jackson Laboratories) male and female mice were used for these experiments. C57BL/6 mice (Sham: *n* = 5 males and *n* = 3 females; single mTBI: *n* = 5 males and *n* = 3 females; two weekly rmTBI: *n* = 5 males and *n* = 3 females; three weekly rmTBI: *n* = 5 males and *n* = 3 females) were selected as they are commonly used in TBI research and facilitate the use of the TCRβ^−/−^ δ^−/−^ (Sham: *n* = 2 males and *n* = 2 females; three weekly rmTBI: *n* = 2 males and *n* = 2 females) T cell knockout model. The age of the mice was determined by the higher TBI rate among juveniles, which is 1591.5 per 100,000.^3^

Animals were housed five per cage with free access to food and water and a 12-h light/dark cycle. The design of the experiments, housing, handling, care, and processing of the animals were conducted following the Guide for the Care and Use of Laboratory Animals and the Animal Welfare Act, and all experimental procedures were approved by the Virginia Commonwealth University (#AD10003619) Institutional Animal Care and Use Committees.

### Impact acceleration (weight drop) injury

**FIG. 1.**
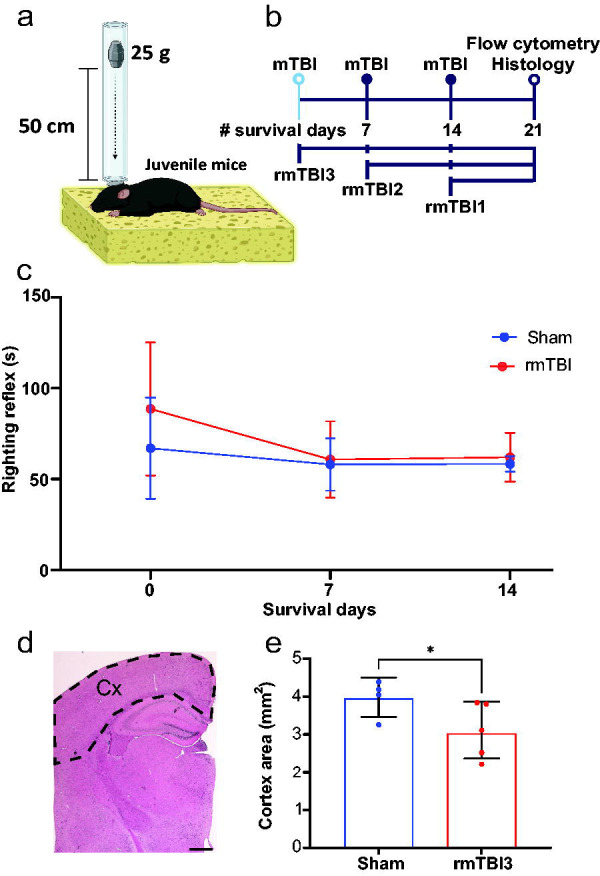
Experimental design and righting reflex recovery. **(a)** On survival day 0, the impact acceleration injury was induced by dropping a 25 g weight at a height of 50 cm onto the steel disk placed on the head of the mice. **(b)** The injury was repeated at a weekly frequency for a total of 3 weeks. One week after the last injury, mice were euthanized, and flow cytometry and histology were performed. **(c)** Quantification of the righting reflex after rmTBI over three consecutive weeks. **(d)** Representative image of hematoxylin staining in the cortex. **(e)** Quantification of cortical area. Mean values are plotted ± standard deviation. Unpaired *t*-test was used to determine statistical differences; *n* = 4–5 mice per group. Scale bar = 400 μm. mTBI, mild traumatic brain injury; rmTBI, repetitive mild traumatic brain injury.

Juvenile mice, at P21, were injured using a modified Marmarou model^19–21^ of impact acceleration that induces a diffuse TBI. Mild injury severity was achieved by adjusting the mass of the impact weight. When a 50 g weight was used on *n* = 2 mice, injury was lethal after the first impact, leading to the selection of a 25 g weight for subsequent experiments. The rmTBI impact acceleration injury was performed weekly for three consecutive weeks. Each injury was produced as follows. The animal was placed in an anesthesia induction chamber and anesthetized for 5 min with 4% isoflurane gas. Then, the mouse was removed and placed prone on a foam bed (Fig. 1a). This paradigm, in contrast to stereotactic fixation, allows for rapid acceleration and deceleration movement of the head during impact injury. A flat stainless steel disc (1.3 cm × 1 mm thick, 880 mg) was placed in the center of the animal’s head. The disc serves to diffuse the acceleration impact across the head and prevent skull fracture. The mouse was positioned under a vertical Plexiglass weight drop tube containing a 25 g weight suspended via a pin, 50 cm above the disc. Impact injury was initiated by pulling the pin and allowing the weight to free fall and collide with the disc on the mouse’s head while the mouse rested on a foam pad. Following injury, the unconscious animal was positioned supine and placed on a sterile polyline absorbent towel for recovery on a heating pad. The righting reflex recovery time was ascertained as a measure of loss of consciousness.^22^ Following the return of reflexes, the mouse was allowed to recover for at least 45 min in a clean cage that had been warmed on a heating pad. Sham animals underwent the same procedure as the rmTBI mice (including three anesthetic exposures and placement under the Plexiglas tube) without any actual impact. Randomly assigned groups of animals were sacrificed for flow cytometry and histology at 21 days after the first impact.

### Flow cytometry cell isolation

Cell suspensions were obtained as previously described.^23^ Blood and targeted brain tissue (specifically, the complete cortex including white matter) from one cerebral hemisphere were collected for analysis 21 days following the initial impact acceleration injury. Mice were anesthetized with isoflurane, and 25 μL of blood was collected from the retro-orbital sinus in ethylenediaminetetraacetic acid (EDTA) tubes immediately before transcardial perfusion with ice-cold 0.1 M heparinized phosphate-buffered saline (PBS) for 3 min. The complete cortical tissue, including white matter, from one hemisphere was dissected, and all the remaining brain tissue such as thalamus, striatum, and hippocampus was discarded. The dissection was performed on ice and digested at 37°C for 15 min with collagenase D (400 units/mL; Roche Diagnostics GmbH, Mannheim, Germany) in Dulbecco’s PBS (Lonza, Basel, Switzerland), each containing 50 μg/mL of DNase I (Sigma-Aldrich, St Louis, MO). The tissue was then mechanically dissociated with a glass Pasteur pipette, filtered through a 70-μm nylon cell strainer, and centrifuged at 300 *g* for 15 min. A 25% Percoll (Sigma-Aldrich) column was used to remove cell debris and myelin, followed by centrifugation at 300 *g* for 10 min. A 25 μL blood sample was mixed with 1× Red Blood Lysis Buffer (Roche) and incubated in rotation for 15 min at room temperature (RT). Samples were then centrifuged at 400 *g* for 5 min at RT. The supernatant was discarded, and cells were washed and resuspended in 1 mL of cytometer buffer (0.5% bovine serum albumin [Sigma-Aldrich], 5 mM EDTA [Millipore, Burlington, MA] in PBS). Then the cells from blood and brain samples were resuspended in 100 μL of cytometer buffer and stained.

### Flow cytometry staining and analysis

Cells were incubated for 5 min at RT with Zombie NIR Dye (1:2000, BioLegend, San Diego, CA) to assess their viability. The Zombie NIR Dye was quenched, and cells were washed with cytometry buffer and blocked with FcR blocking reagent (1:50; Miltenyi Biotec, Bergisch Gladbach, Germany) for 15 min. Then the samples were washed with cytometry buffer and stained with antibodies (Table 1), fixed, and permeabilized by using the FoxP3/transcription factor staining buffer set (eBiosciences, Waltham, MA) following the manufacturer’s instructions. Briefly, cells were fixed for 7 min at 4°C, washed, permeabilized, and stained with intracellular markers (Table 1) for 30 min at 4°C. This panel was titrated and optimized with all surface markers using 0.5 μL of each antibody and fluorescence minus one control. All samples were stained in the presence of Brilliant Stain Buffer (563794, BD Bioscience), per the manufacturer’s protocol. Then, cells were washed and analyzed on a Cytek Aurora Spectral flow cytometer (Cytek Biosciences, Fremont, CA) using SpectroFlo software. Data were analyzed using FlowJo Software Version 10.10.

**Table 1.** Antibodies Used in the Study

Target	Fluorophore	Main laser	Clone	Catalog number	Company
CD45	BUV395	UV	30-F11	bdb564279	BD (Fisher)
F4/80	BUV496	UV	T45-2342	bdb750644	BD (Fisher)
CD4	BUV737	UV	GK1.5	bdb612761	BD (Fisher)
MHC-II	eFluor 450	Violet	M5/114.15.2	48-5321-82	eBiosciences
Tcrgd	BV786	Violet	GL3	bdb744117	BD (Fisher)
CD206	PerCP-eFlour 710	Blue	MR6F3	46-2061-82	eBiosciences
CD11b	PE/Fire640	Yellow/Green	M1/70	101280	BioLegend
TCRb	AF700	Red	H57-597	109224	BioLegend
Live/Dead	Zombie NIR	Red		423106	BioLegend
CD8	APC/Fire 750	Red	53-6.7	100765	BioLegend
GFAP	Cy3			C9205	Sigma-Aldrich
Iba1				NC9288364	Fisher Scientific

GFAP, glial fibrillary acidic protein; UV, ultraviolet.

### Tissue processing

As described above, transcardial perfusion was performed with PBS. One hemisphere was used for flow cytometry, and the other hemisphere was immersed in 4% paraformaldehyde (Sigma-Aldrich) in PBS (0.1 M, pH 7.2) for 48 h at 4°C. Half brains were embedded in 5% agar (Sigma-Aldrich). Coronal sections of 60 μm thickness were obtained using a vibratome (VT1000 S Vibrating blade microtome; Leica Biosystems, Deer Park, IL).

### Fluorescence immunohistochemistry and histology

Fluorescence immunohistochemical staining was performed on free-floating 60 μm sections. Tissue was blocked for 45 min using blocking solution (PBS with 1% bovine serum albumin and 0.3% Triton X-100 [Sigma-Aldrich]) and stained using glial fibrillary acidic protein (GFAP; 1:1000, mouse, Cat# C9205, Sigma-Aldrich) and Iba1 (1:500, rabbit, Cat# NC9288364, Wako Fisher Scientific) primary antibodies overnight at RT. Sections were then incubated with anti-rabbit 488 (1:1000; Fisher Scientific, Waltham, MA) secondary antibody for 2 h in blocking solution. Tissue sections were removed from solution, washed in PBS, and incubated in 4ʹ,6-diamidino-2-phenylindole (1:5000; Life Technology, Carlsbad, CA) at RT, in the dark, for 10 min. Sections were placed on glass slides, dried, and cover-slipped with Fluoromount-G mounting medium (Thermo Fisher Scientific, Waltham, MA).

For histology, brain sections were stained with hematoxylin (Harris, Biocare Medical, CA, US, 061920A2) for 7 min, then sections were rinsed for 5 min in running tap water and differentiated via a quick dip in 0.5% acid ethanol. 0.5% eosin (Sigma, 1.09844.1000) was applied for 3 min, and sections were dehydrated and mounted in xylene-based medium (Epredia™ Cytoseal™ XYL Mountant, 8312-4).

### Quantitative fluorescent immunohistochemistry

Immunofluorescence images (1024 × 1024 pixel resolution were obtained with a BZ-X800 slide scanning fluorescence microscope (Keyence Corporation of America, Itasca, IL) using a 20× objective for lateral CC and a 10× objective for cortical layers II/III and IV. The number of astrocytes and microglia/macrophages was quantified by counting the number of cells divided by the area of the region of interest (lateral CC, Fig. 5a and cortical layers II/III and IV), and cortical area was measured in four slices spaced 300 μm apart by a blinded observer using ImageJ Fiji software. For each experiment, images were obtained in batches using the same settings. Figures were composed using Adobe Illustrator (Mountain View, CA).

### Statistical analysis

Blinding of investigators to experimental groups was maintained until data were fully analyzed. Data were assessed for normal distribution with the Shapiro–Wilk test and expressed as mean ± standard deviation (SD). One-way analysis of variance (ANOVA) and Dunnett’s multiple comparisons test or unpaired *t*-test were used to compare consecutive weekly injuries. For TCR KO and WT weekly rmTBI conditions, two-way ANOVA and Dunnett’s multiple comparison *post-hoc* test were used. All analyses were performed with GraphPad Prism v10.1.0 (GraphPad Software, Boston, MA). *p* < 0.05 based on both tests was considered statistically significant. Power analysis was performed from the infiltration of CD4^+^ T cells preliminary data, and based on independent *t*-tests, the estimated power for each group comparison is as follows: sham versus 1mTBI: power achieved = 0.900; sham versus 2mTBI: power achieved = 0.603; sham versus 3mTBI: power achieved = 0.997.

## Results

### Diffuse weekly rmTBI did not induce an increase in the righting reflex time

To induce diffuse rmTBI, we employed a weekly impact acceleration model calibrated for mild subconcussive TBI^19–21,24^ using juvenile mice of both sexes at P21 (Fig. 1a). The impact occurred once every 7 days for 3 weeks, after which the animals were euthanized 21 days following the first impact (Fig. 1b). Additionally, weekly rmTBI did not result in mortality.

Previous reports have demonstrated a deleterious effect, showing axonal damage despite lack of alteration in responsiveness following a single subconcussive mTBI by modifying the Marmarou model.^25–27^ Thus, we initially assessed the righting time recovery reflex, comparing the three weekly rmTBI groups to the sham group (Fig. 1c). Isoflurane anesthesia caused a brief period of unconsciousness; however, no significant changes in righting reflex were observed between any of the injury groups across the three time points (Fig. 1c). When cortical area was analyzed, a significant decrease was detected when comparing shams to rmTBI3 group (Fig. 1d).

### Weekly rmTBI is associated with infiltration of CD4^+^/CD8^+^ T cells and macrophages

**FIG. 2.**
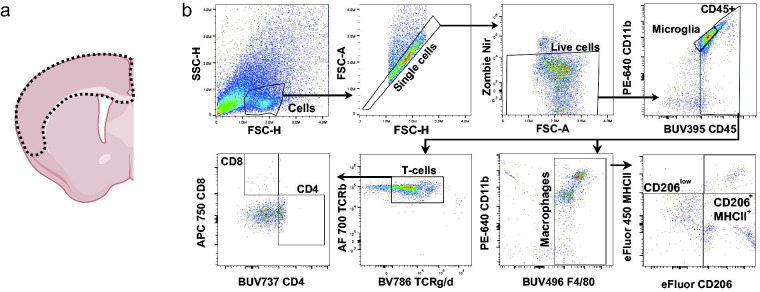
Brain tissue used and gating strategy for the immunotyping panel. **(a)** Schematic representation of the brain tissue used for the flow cytometry analysis. **(b)** Gating strategy used for the detection of the infiltrating immune cells.

**FIG. 3.**
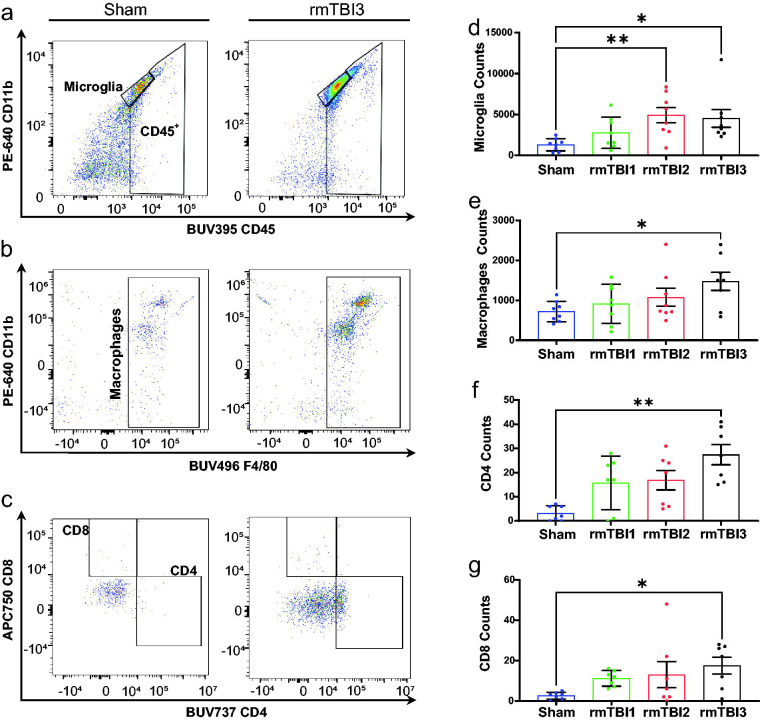
Three consecutive weekly rmTBI in juvenile mice increased the infiltration of peripheral immune cells. Gating strategy for **(a)** CD45lowCD11b^+^ microglia, **(b)** CD11b^+^ F4/80^+^ macrophages, and **(c)** CD8^+^ and CD4^+^ T cells. Quantification of **(d)** CD45lowCD11b^+^, **(e)** CD11b^+^ F4/80^+^ macrophage, **(f)** CD4^+^, and **(g)** CD8^+^ total cell counts. Mean values are plotted ± standard deviation. One-way ANOVA and Dunnett’s multiple comparisons test were used to determine statistical differences. **p < 0.001, *p < 0.05; *n* = 8–7 mice per group. ANOVA, analysis of variance; rmTBI, repetitive mild traumatic brain injury.

TBI-induced neuroinflammation is a significant contributor to chronic neurodegeneration and functional deficits^28–30^ and has been linked to the infiltration of peripheral T lymphocytes and macrophages.^31,32^ However, the role of infiltrating immune cells in the severity of rmTBI during brain development remains unclear. We therefore investigated peripheral immune cell infiltration into the brain parenchyma (Fig. 2a) in our pediatric rmTBI model using a flow cytometry immunophenotyping panel detection approach (Fig. 2b). Overall, the survival rate of the isolated brain cells was 98.15% (±SD 1.9) and 89.8% (±SD 3.04) of live blood cells. Our results showed an enrichment in infiltrating peripheral immune cells of mice with rmTBI compared to sham, including CD45^low^ CD11b^+^ microglia (Fig. 3a), CD11b^+^ F4/80^+^ macrophages (Fig. 3b), and CD4^+^CD8^+^ T cells (Fig. 3c). Our data revealed a distinct, impact-dependent pattern of immune cell infiltration in the brain following rmTBI. CD45^low^ CD11b^+^ microglia increased after the second weekly impact and remained high through the consecutive impact (Fig. 3d). Furthermore, CD11b^+^ F4/80^+^ macrophage, CD8^+^, and CD4^+^ T cells showed a gradual increase with the number of impacts, showing a significant increase after the third weekly rmTBI (Fig. 3e–g). Notably, the infiltration of CD45^+^ immune cells, CD11b^+^ F4/80^+^ macrophages, CD4^+^ T cells, and CD8^+^ T cells (Fig. 3e–g) was only significantly different after the third weekly rmTBI in juvenile mice compared to sham controls. No significant changes were found between groups when males and females were compared (Table 2). These findings suggest a coordinated and time-dependent immune response in the brain after three weekly rmTBI, highlighting potential windows for targeted immunomodulatory therapies.

**Table 2.** Cell Counts ± Standard Deviation in Females and Males

Biological sex	Group	Macrophages	Microglia	CD4	CD8
Males	Sham	644.6 ± 244.3	1324 ± 929.5	1.750 ± 2.870	3.750 ± 2.500
	rmTBI1	891.2 ± 459.8	2447 ± 1705	9.500 ± 9.740	11.00 ± 3.916
	rmTBI2	1263 ± 761.1	4601 ± 3151	20.75 ± 19.67	14.00 ± 22.80
	rmTBI3	1286 ± 728.4	5494 ± 4320	31.25 ± 31.30	16.50 ± 9.147
Females	Sham	845.0 ± 271.8	1042 ± 586.8	5.000 ± 2.640	1.333 ± 0.577
	rmTBI1	955.0 ± 641.0	2447 ± 1705	21.00 ± 12.49	11.33 ± 4.726
	rmTBI2	772.7 ± 88.12	5078 ± 2893	15.67 ± 13.58	11.67 ± 10.02
	rmTBI3	1788 ± 352.8	3650 ± 960.5	25.67 ± 10.07	21.33 ± 17.95

rmTBI, repetitive mild traumatic brain injury.

### Peripheral blood showed enrichment of immune cells earlier than the brain parenchyma after weekly rmTBI

**FIG. 4.**
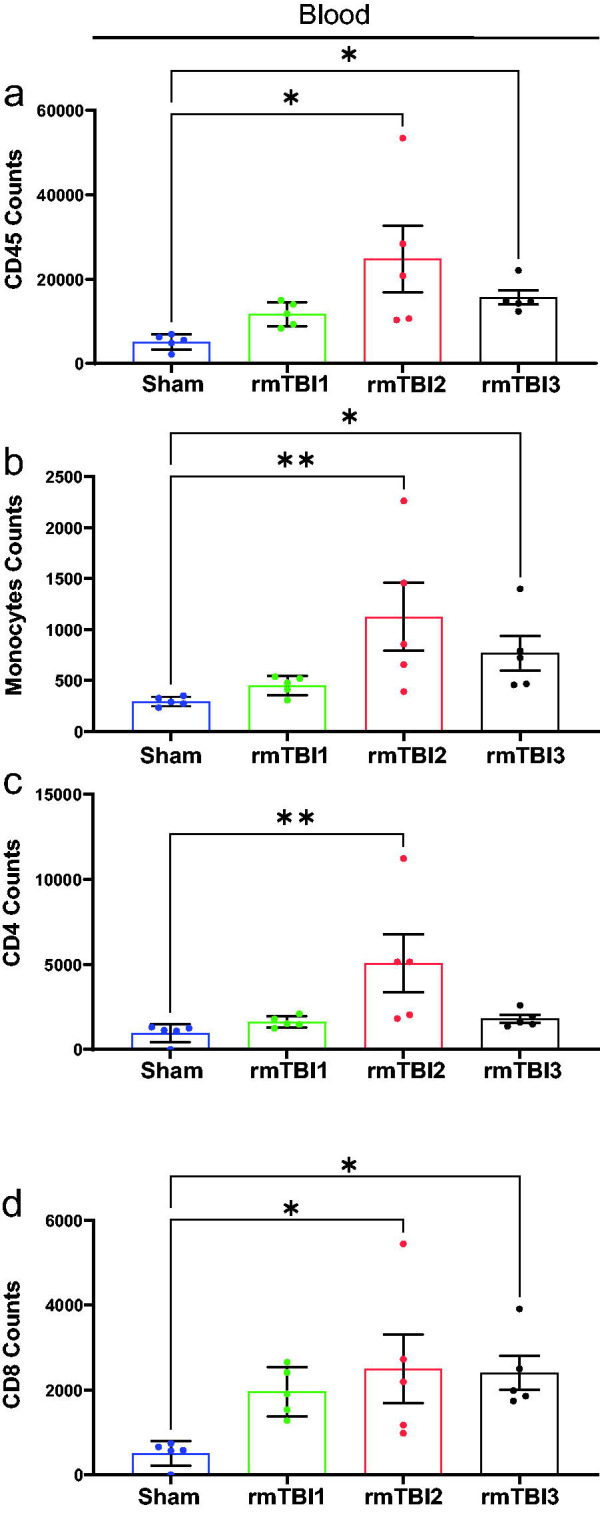
Two weekly rmTBI in juvenile mice increased the immune cells in the peripheral blood. Quantification of **(a)** CD45+ immune cells, **(b)** CD11b^+^ F4/80^+^ monocytes, **(c)** CD4^+^ T, and **(d)** CD8^+^ T total cell counts. Mean values are plotted ± standard deviation. One-way ANOVA and Dunnett’s multiple comparisons test were used to determine statistical differences. **p < 0.001, *p < 0.05; *n* = 5 mice per group. ANOVA, analysis of variance; rmTBI, repetitive mild traumatic brain injury.

We analyzed the presence of the same immune cell populations (CD45^+^, CD11b^+^ F4/80^+^ monocytes/macrophages, and CD4^+^/CD8^+^ T cells) in the blood of the injured mice (Fig. 4). The impact-dependent pattern of immune cells differed between the blood and brain parenchyma following rmTBI. We observed an increase in CD45^+^ (Fig. 4a), CD11b^+^ F4/80^+^ monocytes (Fig. 4b), CD4^+^ T cells (Fig. 4c), and CD8^+^ T cells (Fig. 4d) after the second weekly rmTBI compared to sham controls. This observation suggests that these peripheral immune cells may be primed following the second rmTBI and begin to infiltrate the brain parenchyma after the third injury.

### Juvenile mice showed an increase in neuroinflammation in the lateral CC after weekly rmTBI

**FIG. 5.**
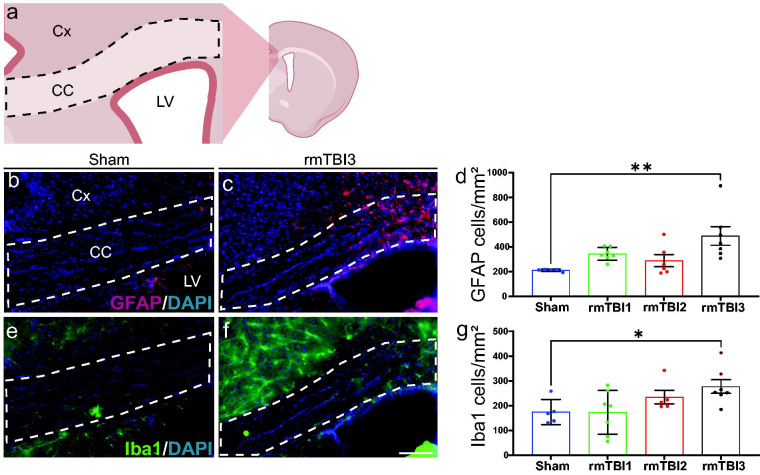
Juvenile mice subjected to three weekly rmTBI exhibited an increased density of astrocytes and microglia/macrophages in the lateral CC. **(a)** Schematic representation of the region of interest. **(b, c)** Representative images of GFAP staining in the lateral CC. **(d)** Quantification of GFAP+ cell density in the lateral CC. **(e, f)** Representative images of Iba1 staining in the lateral CC. **(g)** Quantification of Iba1 cell density in the lateral CC. Mean values are plotted ± standard deviation. One-way ANOVA and Dunnett’s multiple comparisons test were used to determine statistical differences. **p < 0.001, *p < 0.05; *n* = 5–7 mice per group. Scale bar = 100 μm. ANOVA, analysis of variance; CC, corpus callosum; Cx, cortex; GFAP, glial fibrillary acidic protein; LV, lateral ventricle; rmTBI, repetitive mild traumatic brain injury.

**FIG. 6.**
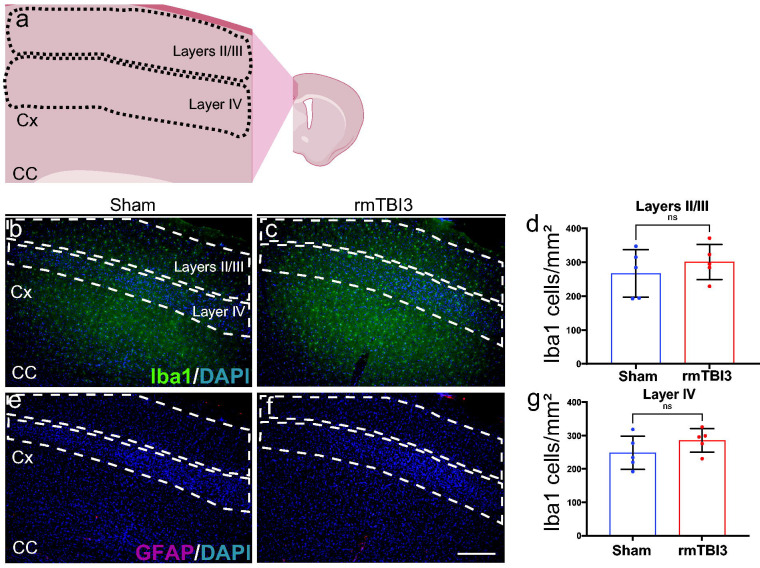
Juvenile mice subjected to three weekly rmTBI did not show microglia/macrophages in the cortical layers II, III, and IV. **(a)** Schematic representation of the region of interest. **(b, c)** Representative images of GFAP staining in the cortex. **(d)** Quantification of Iba1^+^ cell density in layers II and III. **(e, f)** Representative images of Iba1 staining in the cortex. **(g)** Quantification of Iba1^+^ cell density in the cortex. Mean values are plotted ± standard deviation. Unpaired *t-*test was used to determine statistical differences; *n* = 5 mice per group. Scale bar = 100 μm. CC, corpus callosum; Cx, cortex; GFAP, glial fibrillary acidic protein; rmTBI, repetitive mild traumatic brain injury.

One potential mechanism contributing to neurological deficits following TBI is the dysregulation of inflammatory responses.^33^ After assessing the infiltration of immune cells into the brain parenchyma, we further investigated how rmTBI affects the neuroinflammatory response in the lateral CC of juvenile mice (Fig. 5a), we analyzed the staining of GFAP^+^ astrocytes (Fig. 5b and c) and Iba1^+^ microglia/macrophages (Fig. 5e and f) in the lateral CC following one, two, and three weekly rmTBI. Our findings revealed that, after three weekly rmTBI impacts, juvenile mice exhibited increased neuroinflammation in the lateral CC, as indicated by a rise in cell density of GFAP^+^ astrocytes (Fig. 5d) and Iba1^+^ macrophages/microglia (Fig. 5g) compared to the sham group. Similar to the infiltration of immune cells described above, there was a gradual increase in astrocyte and microglia/macrophages density with consecutive injury (Fig. 6d and g). When the density of Iba1^+^ cells in cortical layers II, III, and IV (Fig. 6a) was analyzed (Fig. 6b and c), no significant differences in density were detected (Fig. 6d and g). GFAP^+^ astrocytes were not commonly observed in the cortical tissue (Fig. 6e and f). This suggests that the infiltration of peripheral immune cells may exacerbate neuroinflammation in the lateral CC and not in the cortical gray matter after three consecutive weekly rmTBI impacts in the juvenile mouse brain.

### Juvenile TCRβ^−/−^ δ^−/−^ mice exhibited reduced infiltration of CD206^low^ macrophages after three weekly rmTBI

**FIG. 7.**
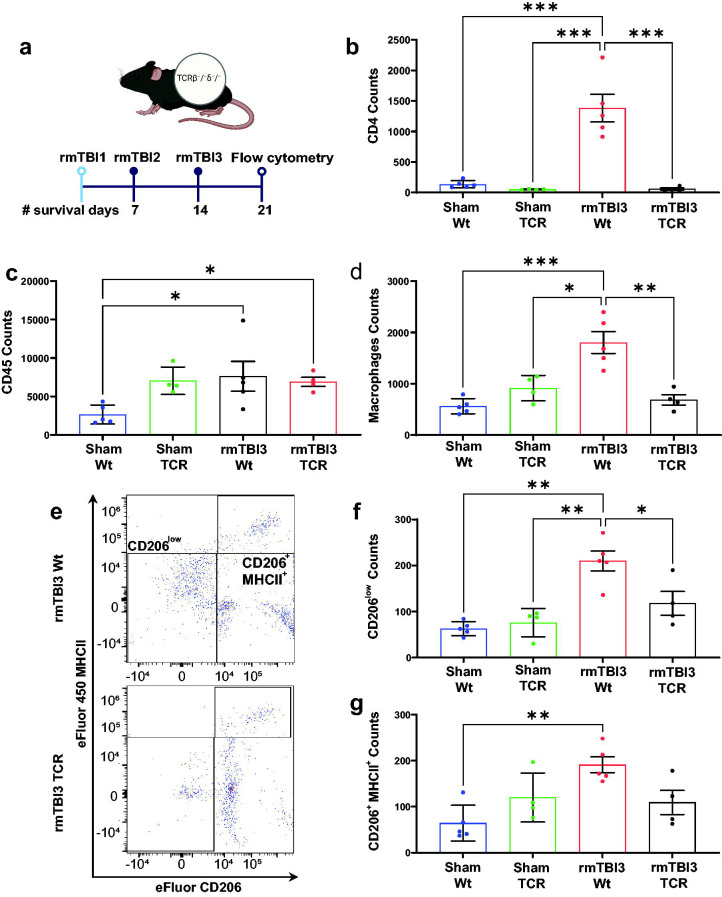
Absence of T cells reduces the infiltration of M1 macrophages after three weekly rmTBI in juvenile mice. **(a)** Experimental design. Quantification of **(b)** CD4^+^, **(c)** CD45^+^, and **(d)** CD11b^+^ F4/80^+^ macrophages total cell counts. **(e)** Gating strategy for pro-inflammatory (CD11b^+^ F4/80^+^ MHCII^−^ CD206^low^) and pro-regenerative (CD11b^+^ F4/80^+^ MHCII^+^ CD206^+^) macrophages. **(f)** Quantification of CD206^low^ total cell count. **(g)** Quantification of CD206^+^ MHCII^+^ total cell count. Mean values are plotted ± standard deviation. Two-way ANOVA followed by Dunnett’s multiple comparisons test, *p < 0.05, ***p < 0.001; *n* = 4–5 mice per group. ANOVA, analysis of variance; rmTBI, repetitive mild traumatic brain injury.

Previous studies have demonstrated that depletion of T cells, using monoclonal anti-CD3 antibodies and TCRβ^−/−^ δ^−/−^ mice in a controlled cortical impact (CCI) model of TBI, prevented chronic neuropathology, neurobehavioral impairments, and myelin degeneration.^34,35^ To further investigate the mechanistic relationship between peripheral T cell infiltration and neuroinflammation in our rmTBI juvenile model, we utilized juvenile TCRβ^−/−^ δ^−/−^ mice subjected to three weekly rmTBI (Fig. 7a). We first confirmed the absence of T cells, including CD4^+^ T cells, in the TCRβ^−/−^ δ^−/−^ comparing shams and rmTBI (Fig. 7b). In the absence of T cells, we still observed a significant increase in the infiltration of CD45^+^ peripheral immune cells (Fig. 7c). In contrast, the absence of T cell prevented the increased CD11b^+^ F4/80^+^ macrophages after rmTBI (Fig. 7d). Notably, when we differentiated between CD206^low^ and CD206^+^ MHCII^+^, CD11b^+^ F4/80^+^ macrophages (Fig. 7e), we found a decrease in CD206^low^ pro-inflammatory macrophages (Fig. 7f) and no significant differences in MHCII^+^ CD206^+^ pro-regenerative macrophages (Fig. 7g) in the TCRβ^−/−^ δ^−/−^ group compared to wild-type mice following the repetitive injury. Our data suggest that T cells play a role in modulating the activation of infiltrating macrophages under the condition of rmTBI in juvenile mice.

### Juvenile TCRβ^−/−^ δ^−/−^ mice presented reduced neuroinflammatory response in the lateral CC after three weekly rmTBI

**FIG. 8.**
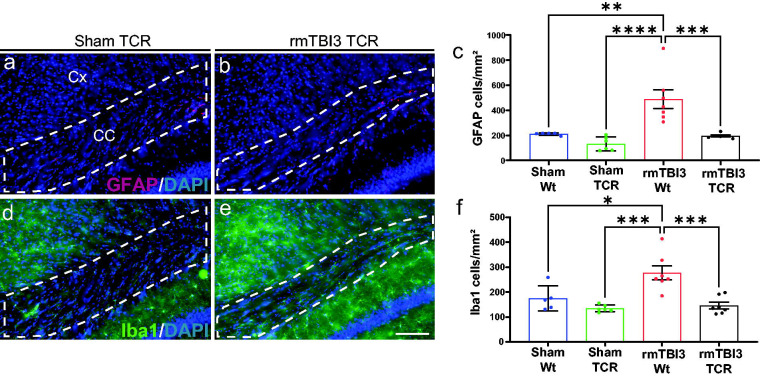
Absence of T cells prevented neuroinflammation in the lateral CC of juvenile mice after three weekly rmTBI. Representative images of GFAP staining in the lateral CC of **(a)** sham and **(b)** injured mice. **(c)** Quantification of GFAP^+^ cell density in the lateral CC. Representative images of Iba1 staining in the lateral CC of **(d)** sham and **(e)** injured mice. **(f)** Quantification of Iba1^+^ cell density in the lateral CC. Mean values are plotted ± standard deviation. Two-way ANOVA followed by Dunnett’s multiple comparisons test, *p < 0.05, ***p < 0.001, ***p < 0.0001; *n* = 5–7 mice per group. Scale bar = 100 μm. ANOVA, analysis of variance; CC, corpus callosum; Cx, cortex; DAPI, 4ʹ,6-diamidino-2-phenylindole; GFAP, glial fibrillary acidic protein; rmTBI, repetitive mild traumatic brain injury.

Knowing that T cell absence reduces the infiltration of CD11b^+^ F4/80^+^ CD206^low^ pro-inflammatory macrophages, we aimed to further investigate the neuroinflammatory response in the lateral CC of TCRβ^−/−^ δ^−/−^ mice after three weekly rounds of rmTBI (Fig. 8). We found significant decrease in the density of GFAP^+^ astrocytes (Fig. 8a–c) and Iba1^+^ macrophages/microglia (Fig. 8d–f) in the lateral CC of both sham and injured TCRβ^−/−^ δ^−/−^ juvenile mice compared with rmTBI3 WT group. These results suggest that T cells play a crucial role in the neuroinflammatory response of the developing brain in the context of rmTBI.

## Discussion

This report provides evidence that infiltrating T cells induce neuroinflammation in the developing brains of juvenile mice following rmTBI. When P21 mice were subjected to three consecutive weekly subconcussive brain injuries, we observed infiltration of peripheral CD11b^+^ F4/80^+^ macrophages and CD4^+^ and CD8^+^ T cells. This was accompanied by increased neuroinflammatory markers, specifically GFAP and Iba1, in the lateral CC. Furthermore, our data suggest that T cells play a crucial role in the infiltration of pro-inflammatory macrophages and contribute to the increased density of GFAP^+^ astrocytes and Iba1^+^ macrophages/microglia in the lateral CC. Consistent with previous findings in adult mice using the CCI TBI model, where depleting T cells prevented functional impairments, neuronal loss, reduced monocyte infiltration, and myelin disruption,^34,35^ our study shows that T cells can modulate neuroinflammation in a developing brain after rmTBI. Therefore, our findings suggest that targeting T cells represents a promising immunomodulatory therapeutic avenue for young patients experiencing rmTBI, potentially leading to improved outcomes.

The weekly impact acceleration model used in this study can be adjusted to produce a spectrum of injury severities, ranging from mild to severe, resulting in diffuse axonal injury and loss of consciousness.^19–21^ When calibrated to induce subconcussive injury, this model elicits symptoms such as increased anxiety, sleep disturbances, and cognitive deficits, which closely resemble those reported by human patients with mTBI.^24^ In our study, weekly repetitive impact acceleration, using a 25 g weight dropped from a height of 50 cm, did not cause increased righting reflex compared to shams. However, when this subconcussive injury protocol was administered over three consecutive weekly sessions, we observed significant neuroinflammation within the lateral CC and infiltration of immune cells in the cortex. Thus, this model effectively mimics subconcussive rmTBI in juvenile patients.

Previous studies have demonstrated that TBI induces the infiltration of both adaptive and innate immune cells.^36,37^ However, the role of peripheral immune cells infiltrating into the brain parenchyma in pediatric rmTBI is not well understood. Following single-impact TBI, macrophages infiltrate the brain within 1–2 days and influence microglia activation in adult mice.^38,39^ These findings were not associated with alterations in the T cell response after TBI.^39^ Furthermore, closed head injury in a T and B cell genetic depletion model did not affect the injury severity outcomes 1 week after TBI.^40^ These findings suggest that T cells may not play a role in the modulation of neuroinflammation in the context of adult TBI. However, the role of T cells in neurological outcomes could be influenced by the injury severity and time after TBI. In a CCI model of TBI, T cells were associated with anxiety-like behavior and memory loss at 12 months after injury.^41^ Here, we demonstrate that the pediatric weekly rmTBI model produces an infiltration of peripheral immune cells such as macrophages and CD4^+^/CD8^+^ T cells 1 week after the last impact acceleration injury. This infiltration of peripheral immune cells may be related to the proliferation of astrocytes and microglia/macrophages in the lateral CC of the injured mice. These data suggest that the developing brain could be susceptible to T cell-driven neuroinflammation under weekly rmTBI conditions.

TBI has been associated with long-lasting cognitive dysfunction produced by persistent neuroinflammation, particularly in the developing brain, which is especially vulnerable to cognitive impairments and is more likely to develop long-term academic difficulties.^5,8,9^ These neurobehavioral outcomes could be related to neuronal damage produced by the persistent presence of T cells. This has been described in other neurological diseases such as Parkinson’s disease,^42^ multiple sclerosis,^43^ and amyotrophic lateral sclerosis.^44^ Pharmacological depletion of CD8^+^ T cells during the chronic phase after TBI is neuroprotective in adult mice, with improved motor functions and reduced remote spinal cord damage.^16^ Furthermore, in an ischemia–perfusion model, the absence of T cells was protective, with reduced lesion volumes and neurological deficits.^45^ In this article, we demonstrate for the first time that the absence of T cells in juvenile mice resulted in a reduction of infiltration of CD11b^+^ F4/80^+^ MHCII^+^ CD206^low^ pro-inflammatory macrophages after three weekly rmTBI. Furthermore, the genetic depletion of T cells did not induce increased GFAP and Iba1 neuroinflammatory markers in the lateral CC of juvenile mice after rmTBI. This finding suggests that targeting T cells in pediatric rmTBI could potentially mitigate neuroinflammation and enhance neuronal protection.

### Limitations

While the genetically deleted T cells (TCRβ^−/−^ δ^−/−^) model allows for the investigation of T cell roles, it may not fully replicate the complex immune response observed during the acute phase following rmTBI. The absence of T cells could lead to compensatory changes that affect cell interactions and other immune cell populations after the injury. Additionally, this knockout model restricts our ability to explore the role of other T cell subtypes in modulating neuroinflammation. Future research should include pharmacological depletion of specific T cell populations through monoclonal antibodies or conditional knockouts for specific T cell subpopulations. For more accurate identification of peripheral macrophages and microglia, specific markers (e.g., CD163, TREM2, and CX3CR1) will be utilized in future studies. Another significant limitation is the lack of direct analysis of circulating gonadal steroids such as testosterone, estrogen, and progesterone. The juvenile age range used in this study (P21–42) is a period that encompasses pubertal onset in mice. These hormones are known to modulate neuroinflammation and TBI pathology, mediating sex differences and injury outcome.^46–48^ Without individual hormonal measurements, the precise pubertal stage cannot be determined. Consequently, any observed sex-specific lack of differences in TBI pathology cannot be definitively attributed to inherent biological sex mechanisms, as they may be confounded or masked by the unmeasured, differential influence of endogenous gonadal hormones at a specific time after injury. Future studies using this model will include periodic measurements of gonadal hormones to develop diagnostic tools in correlation with the pubertal stage of pediatric patients with rmTBI. Furthermore, we assessed outcomes only one week after the last impact acceleration injury, without observing the chronic changes that may occur at later time points after rmTBI, including neurobehavioral outcomes. The correlation of mouse brain development to human brain age may limit our study. Longitudinal studies could provide a better understanding of the role of T cells in TBI over time.

## Conclusions

This study demonstrates that T cells could modulate the neuroinflammatory response in juvenile mice following three weekly rmTBI. Notably, T cell absence led to decreased pro-inflammatory macrophage infiltration and reduced neuroinflammation in the lateral CC of injured mice. Our findings highlight the impact of repetitive mTBI in the developing brain and the contribution of T cells to the neuroinflammatory response.

## Transparency, Rigor, and Reproducibility

Postnatal day 21 male and female mice of both strains were used for these experiments. Wild-type C57BL/6 mice (Sham: *n* = 5; TBI1: *n* = 7; TBI2: *n* = 5; TBI3: *n* = 7) and T cell knockout TCRβ^−/−^ δ^−/−^ mice (Sham: *n* = 4; TBI3: *n* = 4). No mortality was detected; all the animals were included in this study. The design of the experiments, housing, handling, care, and processing of the animals were conducted in accordance with the Guide for the Care and Use of Laboratory Animals and the Animal Welfare Act, and all experimental procedures were approved by the Virginia Commonwealth University (#AD10003619) Institutional Animal Care and Use Committees. Blinding of investigators to experimental groups was maintained until data were fully analyzed. As previously described in the section “Statistical Analysis,”, data were assessed for normal distribution with the Shapiro–Wilk test and expressed as mean ± SD. One-way ANOVA and Kruskal–Wallis multiple comparisons test or unpaired *t-*test were used to compare consecutive weekly injuries. For T cell knockout and wild-type weekly rmTBI conditions, two-way ANOVA and Dunnett’s multiple comparisons *post-hoc* test were used. *p* < 0.05 based on both tests was considered statistically significant. All analyses were performed with GraphPad Prism v10.1.0 (GraphPad software). Microscopy and imaging data reported in this article will be shared upon request. This article does not report the original code. Any additional information reported in this article is available upon request.

## Authors’ contributions

Conceptualization: K.S., J.G., J.P.M., K.J., and D.D.L.; Methodology: K.S., L.A., Y.S., G.D., R.S., W.B., and M.G.B.; Visualization: K.S. and L.A. Funding acquisition: K.J. and D.D.L. Project administration: K.S., K.J., and D.D.L.; Supervision: J.P.M., K.J., and D.D.L. Writing – original draft: K.S. and L.A. Writing – review and editing: all authors.

## Abbreviations

CC: corpus callosum
CCI: controlled cortical impact
DAPI: 4ʹ,6-diamidino-2-phenylindole
EDTA: ethylene diamine tetraacetic acid
GFAP: glial fibrillary acidic protein
iba1: ionized calcium-binding adapter molecule 1
mTBI: mild traumatic brain injury
PBS: phosphate-buffered saline
rmTBI: repetitive mild traumatic brain injury
RT: room temperature
SD: standard deviation
TBI: traumatic brain injury
